# Peak Oxygen Uptake after Cardiac Rehabilitation: A Randomized Controlled Trial of a 12-Month Maintenance Program versus Usual Care

**DOI:** 10.1371/journal.pone.0107924

**Published:** 2014-09-23

**Authors:** Erik Madssen, Ingerid Arbo, Ingrid Granøien, Liv Walderhaug, Trine Moholdt

**Affiliations:** 1 K.G. Jebsen Center of Exercise in Medicine, Department of Circulation and Medical Imaging, Faculty of Medicine, Norwegian University of Science and Technology, Trondheim, Norway; 2 Department of Pulmonary Medicine, St. Olavs Hospital, Trondheim, Norway; 3 Ålesund Hospital, Ålesund, Norway; 4 Women's Clinic, St. Olavs Hospital, Trondheim, Norway; University of Glasgow, United Kingdom

## Abstract

**Background:**

Exercise capacity is a strong predictor of survival in patients with coronary artery disease (CAD). Exercise capacity improves after cardiac rehabilitation exercise training, but previous studies have demonstrated a decline in peak oxygen uptake after ending a formal rehabilitation program. There is a lack of knowledge on how long-term exercise adherence can be achieved in CAD patients. We therefore assessed if a 12-month maintenance program following cardiac rehabilitation would lead to increased adherence to exercise and increased exercise capacity compared to usual care.

**Materials and Methods:**

Two-centre, open, parallel randomized controlled trial with 12 months follow-up comparing usual care to a maintenance program. The maintenance program consisted of one monthly supervised high intensity interval training session, a written exercise program and exercise diary, and a maximum exercise test every third month during follow-up. Forty-nine patients (15 women) on optimal medical treatment were included following discharge from cardiac rehabilitation. The primary endpoint was change in peak oxygen uptake at follow-up; secondary endpoints were physical activity level, quality of life and blood markers of cardiovascular risk.

**Results:**

There was no change in peak oxygen uptake from baseline to follow-up in either group (intervention group 27.9 (±4.7) to 28.8 (±5.6) mL·kg (-1) min (−1), control group 32.0 (±6.2) to 32.8 (±5.8) mL·kg (−1) min (−1), with no between-group difference, *p* = 0.22). Quality of life and blood biomarkers remained essentially unchanged, and both self-reported and measured physical activity levels were similar between groups after 12 months.

**Conclusions:**

A maintenance exercise program for 12 months did not improve adherence to exercise or peak oxygen uptake in CAD patients after discharge from cardiac rehabilitation compared to usual care. This suggests that infrequent supervised high intensity interval training sessions are inadequate to improve peak oxygen uptake in this patient group.

**Trial Registration:**

ClinicalTrials.gov NCT01246570

## Introduction

Exercise-based rehabilitation in patients with coronary artery disease (CAD) reduces mortality [1]–[4], and cardiorespiratory fitness is a strong, independent predictor of both cardiac- and all-cause mortality in patients with CAD [5], [6]. Therefore, it is important to establish effective exercise programs that patients can adhere to in this patient group. Unfortunately, most beneficial effects from physical activity are lost quite rapidly if regular exercise is discontinued. In line with this, peak oxygen uptake (VO_2peak_) has been found to decline at six months, and more so at 30 months, after discharge from cardiac rehabilitation in patients with CAD [7]. Thus, there is a need for studies to assess interventions that may help patients adhere to regular and effective exercise training after ending a formalized cardiac rehabilitation exercise program [8]. It is known that high intensity interval training (HIIT) is more effective than continuous training with low-to-moderate intensity with respect to increasing VO_2peak_ in patients with CAD [9], but there is a lack of knowledge regarding long-term effects of HIIT interventions.

The primary aim of this study was therefore to assess if a 12-month maintenance program following discharge from formal cardiac rehabilitation would improve adherence to physical activity and peak oxygen uptake We hypothesized that the maintenance program would lead to an attenuated decline in VO_2peak_ 12 months after ending the formal cardiac rehabilitation, compared with patients in usual care. Secondary endpoints were physical activity level, quality of life and blood markers of cardiovascular risk.

## Materials and Methods

### Design

**Figure 1:**
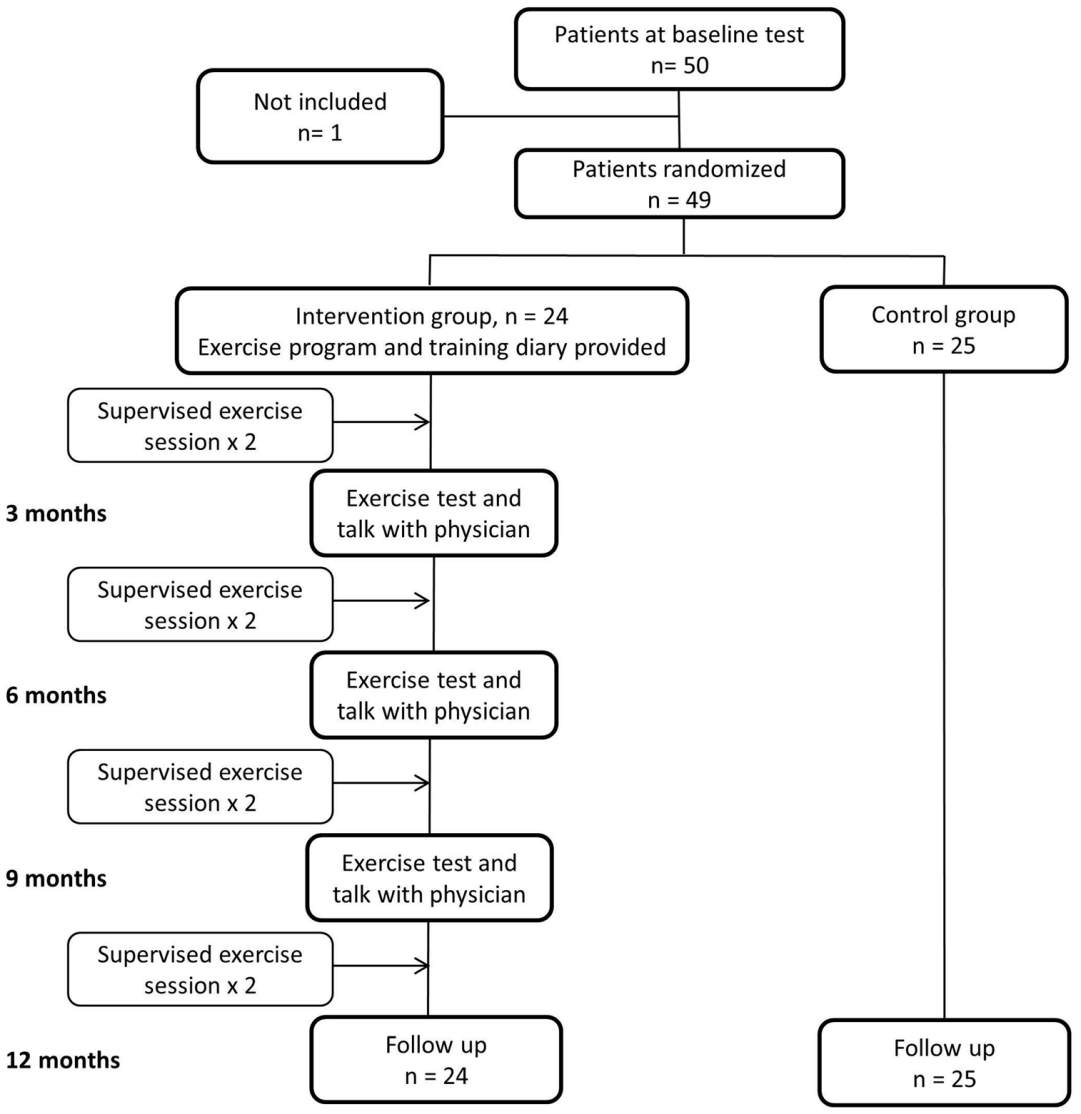
Flow chart of participants throughout the study.

The protocol for this trial and supporting CONSORT checklist are available as supporting information; see Checklist S1 and Protocol S1. This was a two-centre, open, parallel randomized controlled trial. The study was approved by the Regional Committee for Medical and Health Research Ethics in Middle-Norway (REK-Midt 2010/86), and registered at clinicaltrials.gov (NCT01246570). Patients gave their informed, written consent before entering the study, and we performed the study according to the Helsinki declaration for medical research. After acquisition of all baseline data, patients were randomized stratified by centre the same day, using a web-based randomization tool, developed and administered by Unit of Applied Clinical Research, Department of Cancer Research and Molecular Medicine, Norwegian University of Science and Technology, Trondheim. Stratified for study centre, patients were randomized either to a maintenance exercise program or to usual care for 12 months with a 1∶1 allocation ratio (Figure 1).

### Participants

We recruited optimal medically treated patients above 18 years of age from the cardiac rehabilitation centres at St. Olav's University Hospital in Trondheim, and Ålesund Hospital, both in central Norway. Inclusion criteria were completion of a 12-week organized in-hospital cardiac rehabilitation program, consisting of 2 exercise sessions per week, including both HIIT and moderate continuous exercise. Patients were included in the current study 1–2 weeks after ending the formal cardiac rehabilitation program. Patients were excluded if they fulfilled one or more of the following criteria; unstable angina pectoris (chest pain at light physical activity), hemodynamic significant valvular disease (valvular disease confirmed by echocardiography and dyspnoea at light physical activity), chronic obstructive pulmonary disease or chronic heart failure with symptoms at rest or in light physical activity, uncontrolled arterial hypertension (hypertension grade 2 despite medical treatment) chronic renal failure (serum creatinine>140 µmol/L), or pregnancy.

### Interventions

#### Maintenance program group

Patients in the maintenance program group received a written exercise program with the aim of three sessions of high intensity interval training (HIIT) per week, and were invited to attend a monthly supervised exercise session at the hospital. The HIIT program was based on protocols from previous studies [10]–[14], and consisted of 8–10 minutes of warm-up, followed by four times four minutes intervals, with an active pause of three minutes in-between intervals and at the end. The target heart rate was 85–95% of the maximum heart rate as measured at the initial exercise test in intervals, and 70% of maximum heart rate in the active pauses.

During the supervised exercise sessions once per month, patients walked or ran on treadmills (Woodway PPS55, Weil am Rhein, Germany), wearing heart rate monitors (Polar Electro, Kempele, Finland) to ensure that they reached target heart rate. An experienced physiotherapist and/or exercise physiologist instructed and motivated the patients on how to perform HIIT, and patients were asked about their home exercise training and encouraged to maintain a high level of activity. Such instructions have previously been found to be enough to give an acceptable adherence to exercise training during 6 months [10]. The written exercise program also explained in detail how to perform the HIIT, and patients were instructed to perform HIIT as home-based exercise training, as activities which engaged large muscle groups, i.e. walking uphill, running, cross-country skiing, or bicycling. Patients were told to either use a heart rate monitor to ensure that the intensity was adequate, or to exercise with an intensity making them breathe heavily during home exercise training. We gave no advice regarding diet or other lifestyle factors during the follow-up period.

Every third month, patients in the maintenance group did a maximum exercise test as described below. These tests were done to monitor their exercise capacity; to give patients individualized feedback on their VO_2peak_ level, and to ensure them that maximal effort was well tolerated.

#### Control group

Patients in the control group received no instructions in how to exercise apart from what was given by the rehabilitation staff at the hospitals as usual care. In the two participating hospitals, CAD patients are encouraged to be physically active but are not given any concise exercise prescription. The control group attended baseline and 12 months follow-up tests as described below, without any other contact with the study or hospital personnel.

### Outcomes and follow-up

The primary outcome measure was change in VO_2peak_ between baseline and 12 months follow-up. Secondary outcome measures were physical activity level, resting blood pressure, health-related quality of life, cardiometabolic blood markers and anthropometric measurements. In the original study protocol, endothelial function measurements (flow mediated dilatation of the brachial artery) were described. Due to technical issues at one of the participating hospitals, endothelial function could not be included in the study. Physical activity registration was not pre-specified in the original protocol, but during the preparation phase of the study it became clear that such data would be needed and thus physical activity questionnaires and activity monitors were included into the protocol.

#### Peak oxygen uptake

All patients performed a treadmill cardiopulmonary maximum exercise test at baseline and at follow-up after 12 months, without discontinuation of prescribed medication. Gas exchange data was analysed (Oxycon Pro, Jaeger, Hoechberg, Germany) continuously using mixing chamber, and patients were monitored with a 12-lead electrocardiogram during maximum exercise tests. After 10 minutes of warm-up, we individually adjusted a ramp protocol, by increasing the incline and speed of the treadmill, for the test to last 8–12 minutes as recommended [15]. The test was terminated when the patients reported to be exhausted, or if clinical symptoms occurred. Peak oxygen uptake (VO_2peak_) was calculated as the mean of the three highest VO_2_ measurements during the test, each obtained over a 10-sec average. Peak heart rate was recorded at the end of the test and was not compared to age predicted values as the patients were taking betablockers. We did not use a threshold value of respiratory exchange ratio (RER) as a criterion for reaching VO_2peak_, but average RER-values obtained during the tests are outlined in the results. Heart rate recovery was defined as the change in heart rate from the peak heart rate to the heart rate after one minute of rest standing on the treadmill.

#### Physical activity level

All patients reported physical activity level by questionnaires. They were asked how often they exercised (≤1, 2–3, ≥4 sessions per week), the mean duration of each exercise session (<30, 30–45, 45–60,>60 minutes per session), and the mean intensity-level during sessions (light, medium or hard intensity). In a subgroup of patients (n = 18, 37%), everyday physical activity level was measured with an on-body activity monitoring system (Sensewear, Bodymedia Inc., Pittsburgh, PA, USA) worn for 3–7 days during the first 1–3 weeks and repeated during the last 1–3 weeks in the follow-up period. We measured the numbers of steps taken per day, and the time spent in different activity level zones defined by metabolic equivalents (METs). Sedentary time was defined as activity <3 METs, moderate physical activity was defined as activity ≥3 and <6 METs, and vigorous physical activity was defined as ≥6 METs.

#### Resting blood pressure and resting heart rate

Resting systolic and diastolic blood pressure was measured sitting after 10 minutes of rest with a calibrated automated blood pressure monitor (Welch Allyn, Germany). Blood pressure was automatically measured three times by the monitor, and the average value of the two last measurements was used. Resting heart rate was measured, after 10 minutes of rest in supine position, using electrocardiogram. Height was measured to the nearest centimetre and body weight was measured to the nearest 0.1 kg using a Seca 877 scale (Seca Corp, Germany). Body mass index was calculated as body weight in kilograms divided by the square of height in meter. Waist circumference was measured to the nearest half centimetre, using a measuring tape at the height of the umbilicus.

#### Blood markers

Venous blood samples were drawn after 12 hours of fasting, and analysed using accredited in-hospital procedures for serum glucose, total cholesterol, low-density lipoprotein cholesterol, high-density lipoprotein cholesterol, triglycerides, and high-sensitive C-reactive protein (all using standard commercial kits on a Roche Modular P, Roche, Basel, Switzerland), and glycosylated haemoglobin (Roche Cobas Integra 400).

#### Health related quality of life

Health related quality of life was measured by MacNew Heart Disease Health-Related Quality of Life Questionnaire [16]. This is a disease-specific questionnaire with a validated Norwegian translation [17]. The questionnaire assesses social, emotional, and physical quality of life using a 1–7 scale. Higher scores indicated better quality of life and a change of 0.5 is found to be clinically significant [16].

### Sample size and statistics

A decline in VO_2peak_ of 2.0 mL·kg^−1^·min^−1^ (SD 4.0) could be expected after 12 months without formal rehabilitation [18]. We anticipated that the intervention program would give an increase of 2.0 mL·kg^−1^·min^−1^after 12 months, giving a difference of 4.0 mL·kg^−1^·min^−1^ compared to usual care. This yields a standardised difference of 4/4 = 1. With a two-tailed t-test for independent samples at a power (1-β) of 0.9 and α = 0.05, a total of 44 subjects had to be enrolled [19]. To allow for an expected 10% drop out, we aimed at including 48 patients. The actual statistical analysis performed takes within-person correlation into account and thereby improves power.

Observed data are given as frequencies with percentages in parenthesis, or means ± standard deviation (SD). Baseline characteristics were compared using the chi square test, Mann-Whitney U-test or student t-test where appropriate. Changes from baseline to follow-up are reported with 95% confidence intervals (CI) using a univariate general linear model in the analyses. To test for differences between groups, we used a univariate general linear model analysis of covariance (ANCOVA) with Bonferroni adjustments. In the model, the change in the outcome variable (Δ-values) was the dependent variable, with group as fixed factor and baseline values of the outcome variable as covariates [20]. Between-group differences are reported with 95% CIs and p-values. Within- and between-group differences were considered significant if the 95% CI did not include zero [21]. Physical activity data are only presented as descriptive statistics. All statistical analyses were performed using SPSS (version 20.0, IBM, Chicago, IL, USA).

## Results

Forty-nine patients (15 women) were recruited from January 2011 to March 2012 at St.Olav's Hospital and from November 2011 to June 2012 at Ålesund Hospital. Patient characteristics at baseline can be found in Table 1. All registered variables at baseline were comparable between groups except for age, which was lower in the control group (p<0.05, Table 1), and VO_2peak_, which was higher in the control group (p<0.01, Table 2).

**Table 1:** Patient characteristics and medication use at baseline.

	Intervention group (n = 24)	Control group (n = 25)
Sex, male/female	18/6	18/7
Age, years (range)	64.4 (47–78)	58.5 (42–71)*
**Treatment qualifying for referral to rehabilitation**		
PCI	15 (63)	15 (60)
CABG	7 (29)	7 (28)
Valve replacement	2 (8)	1 (4)
Cardiomyopathy	0 (0)	2 (8)
**Co-morbidity**		
Heart failure	3 (13)	4 (16)
PAD	0 (0)	1 (4)
Hypertension	11 (46)	13 (52)
Diabetes	4 (17)	3 (12)
**Medication at baseline**		
Aspirin	24 (100)	25 (100)
Clopidogrel	18 (75)	16 (64)
Warfarin	1 (4)	1 (4)
Betablockers	19 (79)	19 (76)
Statins	23 (96)	25 (100)
ACE/ARA	9 (38)	11 (44)

Data are given as numbers with percentages in parenthesis when not otherwise specified. PCI; percutaneous coronary intervention, CABG; coronary artery bypass grafting, PAD; peripheral artery disease, ACE; angiotensin converting enzyme inhibitors, ARA; angiotensin II receptor antagonists. * Between group difference at baseline (p = 0.02).

**Table 2:** Outcome measures at baseline and after 12 months follow-up.

	Intervention group (n = 24)			Control group (n = 25)			ANCOVA	
	Baseline	12 months	Change scores (95% CI)	Baseline	12 months	Change scores (95% CI)	Between-group difference (95% CI)	P value
*Exercise test*								
VO_2peak_, ml·kg^−1^·min^−1^	27.9±4.7	28.8±5.6	0.9 (−0.6, 2.4)	32.0±6.2	32.8±5.8	0.8 (−0.5, 2.2)	0.6 (−1.5, 2.7)	0.58
VO_2peak_, ml·min^−1^	2405±517	2533±576	129 (−18, 275)	2535±760	2614±734	79 (−45, 203)	−0.04 (−0.2, 0.2)	0.70
RER_peak_	1.09±0.07	1.09±0.09	0.004 (−0.03, 0.04)	1.10±0.07	1.09±0.06	−0.01 (−0.04, 0.01)	−0.01 (−0.05, 0.03)	0.71
HR_peak_, beats	154.7±13.1	155.8±15.9	1.1 (−5.1, 7.3)	160.6±11.3	161.6±13.1	1.0 (−2.3, 4.2)	1.4 (−5.5, 8.3)	0.68
HR recovery, beats	27.7±11.2	31.2±14.6	3.5 (−1.9, 8.9)	28.9±10.0	30.1±10.2	1.2 (−2.9, 5.4)	−1.7 (−8.3, 4.9)	0.60
*Resting measurements*								
HR, beats/min	64.0±10.2	65.7±11.6	1.6 (−1.5, 4.8)	61.2±11.5	63.2±11.1	2.0 (−1.4, 5.4)	−0.2 (−4.8, 4.4)	0.92
Systolic BP, mmHg	132.8±14.7	133.7±16.4	0.9 (−4.4, 6.2)	131.3±14.5	134.3±14.0	3.0 (−2.3, 8.2)	1.4 (−5.9, 8.8)	0.69
Diastolic BP, mmHG	78.8±7.2	79.3±7.5	0.5 (−2.1, 3.1)	75.1±10.7	77.5±10.0	2.4 (−1.5, 6.2)	−0.08 (−4.8, 4.6)	0.97
*Anthropometric measurements*								
Weight, kg	86.8±15.6	89.0±16.5	2.1 (0.8, 3.5)*	79.2±17.8	79.9±17.3	0.7 (−0.8, 2.2)	−1.5 (−3.6, 0.5)	0.14
BMI, kg/m^2^	28.0±3.9	28.7±4.1	0.7 (0.2, 1.1)*	25.8±3.3	26.1±3.2	0.3 (−0.2, 0.7)	−0.5 (−1.2, 0.2)	0.16
Waist, cm	101.4±12.1	103.2±11.7	1.8 (0.1, 3.5)*	92.6±9.8	93.0±9.4	0.3 (−0.8, 1.5)	−2.2 (−4.2, −0.1)‡	0.04
*Quality of life*								
Emotional domain	6.0±0.8	6.0±0.6	0.1 (−0.2, 0.3)	5.7±0.8	6.1±0.8	0.4 (−0.2, 0.7)	0.1 (−0.4, 0.6)	0.69
Physical domain	6.2±0.7	6.3±0.6	0.1 (−0.2, 0.4)	6.3±0.6	6.4±0.5	0.1 (−0.1, 0.3)	0.1 (−0.2, 0.5)	0.40
Social domain	6.4±0.6	6.5±0.4	0.1 (−0.1, 0.3)	6.4±0.6	6.7±0.4	0.3 (0.1, 0.5)*	0.1 (−0.1, 0.4)	0.37
*Blood markers*								
hsCPR, mg/L	1.09±0.9	1.07±0.6	−0.02 (−0.3, 0.3)	1.2±0.9	1.5±2.5	0.3 (−0.7, 1.3)	0.4 (−0.7, 1.4)	0.51
Cholesterol, mmol/L	4.3±1.0	4.3±0.8	−0.08 (−0.4, 0.2)	3.9±0.6	3.9±0.8	−0.02 (−0.2, 0.2)	−0.1 (−0.5, 0.3)	0.57
LDL, mmol/L	2.2±0.9	2.2±0.7	−0.03 (−0.3, 0.2)	2.0±0.5	2.0±0.6	0.01 (−0.2, 0.2)	−0.0 (−0.4, 0.2)	0.70
HDL, mmol/L	1.5±0.4	1.5±0.4	0.05 (−0.02, 0.13)	1.3±0.4	1.3±0.4	0.004 (−0.05, 0.05)	−0.04 (−0.1, 0.04)	0.34
TG, mmol/L	1.4±0.8	1.2±0.7	−0.19 (−0.4, 0.01)	1.2±0.6	1.3±1.1	0.07 (−0.3, 0.4)	0.2 (−0.2, 0.6)	0.24
Glucose, mmol/L	6.7±3.7	6.4±2.2	−0.3 (−0.6, 0.1)	6.0±1.8	6.2±2.4	0.2 (−0.2, 0.6)	0.3 (−0.4, 1.0)	0.43
HbA1c, %	6.1±1.2	6.0±1.0	−0.1 (−0.2, −0.01)*	6.1±0.7	6.2±1.4	0.1 (−0.1, 0.4)	0.2 (−0.1, 0.6)	0.21

VO_2peak_; peak oxygen uptake, RER_peak_; respiratory exchange ratio at peak oxygen uptake, HR_peak_; peak heart rate, HRR, 1 min; heart rate recovery the first minute after ending an exercise test, HR; resting heart rate, SBP; systolic blood pressure, DBP; diastolic blood pressure, BMI; body mass index, hsCRP; high-sensitive C-reactive protein, LDLc; low-density lipoprotein cholesterol, HDLc; high-density lipoprotein cholesterol, TG; triglycerides, HbA1c; glycosylated haemoglobin.

* indicates within-group changes from baseline to 12 months,

‡ indicates between groups changes in mean difference.

We experienced no adverse events during maximum exercise testing or training sessions. All participants completed scheduled tests, and the mean attendance at training sessions was 7.8 out of 8 sessions in the intervention group. None of the patients changed their medication use during the study period. During follow-up, two patients in each group were hospitalized. In the intervention group, one patient was diagnosed with a duodenal ulcer and atrial fibrillation, and one patient was diagnosed with chronic lymphatic leukaemia. In the control group, one patient experienced a tibia fracture and one patient underwent surgical treatment for breast carcinoma.

**Figure 2:**
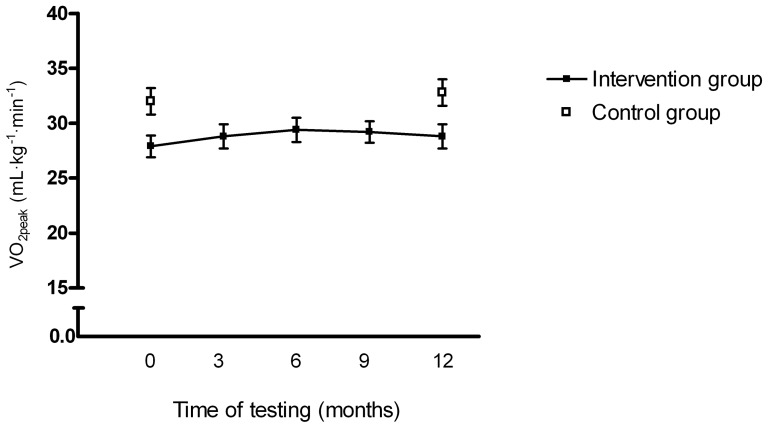
Peak oxygen uptake in the intervention group at baseline, after 3, 6 and 9 months, and at follow-up, and peak oxygen uptake in the control group at baseline and follow-up. The bars represent ± standard error of mean.

We found no within-group changes in VO_2peak_ from baseline to follow-up, and no between-group difference (*p* = 0.22, Table 2). In the intervention group, VO_2peak_ was at its highest at the third maximum exercise test, and declined slightly at test number four and five (Figure 2). We found no changes in resting heart rate, heart rate recovery, blood pressure, neither within- or between-groups from baseline to follow-up. Blood markers did not change during follow-up in any group (Table 2). Quality of life (social domain) was increased in the control group, but there was no between-group difference (*p* = 0.39).

Self-reported physical activity level is summarized in Table 3. In the intervention group, all patients reported some regular physical activity both at baseline and follow-up, whereas 5 patients in the control group reported no regular physical activity at follow-up. The majority of patients in both groups reported that they had been physically active 2–3 times per week, with duration of 45–60 minutes and with a medium intensity. Physical activity level was adequately monitored in 18 patients (37%). The mean number of steps taken per day was 7024 in the intervention group (10 patients), and 9338 in the control group (8 patients). The mean time spent in sedentary, moderate and vigorous activity level zones per day was 1333 minutes, 58 minutes and 3 minutes in the intervention group. The corresponding numbers in the control group were 1288 minutes, 111 minutes, and 7 minutes, respectively.

**Table 3:** Self-reported physical activity.

	Intervention group (n = 24)		Control group (n = 25)	
	Baseline	Follow-up	Baseline	Follow-up
*Self-reported*	n = 19*	n = 21*	n = 22*	n = 22*
**Frequency**				
No exercise	0 (0)	0 (0)	1 (5)	5 (23)
<1/week	1 (5)	0 (0)	0 (0)	0 (0)
1/week	0 (0)	1 (5)	0 (0)	2 (9)
2–3/week	12 (63)	16 (76)	17 (77)	14 (64)
≥4/week	6 (32)	4 (19)	4 (18)	1 (4)
**Duration**				
<30 min	0 (0)	1 (5)	1 (5)	0 (0)
30–45 min	6 (31.5)	6 (28.5)	2 (9)	5 (29.5)
45–60 min	10 (52.5)	8 (38)	11 (50)	5 (29.5)
>60 min	3 (16)	6 (28.5)	7 (36)	7 (41)
**Intensity**				
Low	1 (5)	1 (5)	1 (5)	1 (6)
Medium	16 (84)	11 (52)	13 (62)	10 (59)
High	2 (11)	9 (43)	7 (33)	6 (35)

Data are given as numbers with percentages in parenthesis.

* Valid questionnaires for 19 patients at baseline and 21 patients at follow-up in the intervention group, and 22 patients at baseline and follow-up in the control group.

## Discussion

In this randomized trial we assessed whether patients with CAD discharged from cardiac rehabilitation would benefit with respect to VO_2peak_ by attending a maintenance exercise program for 12 months compared to usual care of no formal follow-up. Our main finding was that the maintenance program did not improve VO_2peak_ compared to usual care. However, neither group deteriorated with respect to exercise capacity during follow-up. This was to us unexpected as exercise capacity has been found to decrease in CAD patients after discharge from formal rehabilitation when the patients were not aware of the last follow-up test [18].

We can only suggest some potential explanations for our findings. Participants enrolled in clinical exercise studies may be highly motivated to perform exercise, especially when they are aware of future follow-up tests. This was illustrated by Gupta et al. [22] who found an increased level of physical activity and 6-minute walking distance at 12 months after discharge from cardiac rehabilitation. Also Izawa et al. [23] demonstrated high exercise maintenance in patients with myocardial infarction 18 months after cardiac rehabilitation. Both these studies had an observational design without strategies to improve exercise adherence after ending formal rehabilitation, and with patients being aware of future follow-ups. We argue that the knowledge of future follow-up tests by cardiac rehabilitation units may act as a motivation for increased levels of physical activity. This may have also been the case in our study. In fact, the objectively measured physical activity indicated that patients in the control group spent more time in the moderate activity level zone and had a higher daily step count than the patients in the intervention group. Patients randomized to the intervention group were on average 6 years older than in the control group, possibly explaining the tendency for lower level of physical activity in the intervention group. Previous studies have found adherence to physical activity to decline with age [24], [25]. However, a previous study found no significant effect of age on improvement in VO_2peak_ after HIIT in patients with CAD [26]. We therefore think that the potential effect of age was on the adherence to exercise rather than on the physiological adaptations to HIIT. Of note, all included patients had some knowledge of HIIT, as this was included in the cardiac rehabilitation program prior to study inclusion.

Several studies have demonstrated that HIIT is superior to moderate continuous exercise in improving VO_2peak_ in patients with CAD [12], [27], [28] without compromising safety [29]. In the current study, patients in the intervention group were invited to attend a monthly supervised HIIT session at the hospital. However, one monthly session of HIIT is obviously not enough to improve or maintain exercise capacity, and therefore patients also received a training program with the aim of three sessions of HIIT per week. A previous study [10] of home-based HIIT after coronary artery bypass grafting has shown good adherence to such exercise prescription. According to the self-reported physical activity data in the current study, however, only about one third of patients in the intervention group reported that they exercised with high-intensity 2–3 times a week. Thus, a lack of adherence to prescribed exercise at home in the maintenance group is probably the single most important explanation to the lack of VO_2peak_ improvement at 12 months.

Our study may have implications for clinical rehabilitation units. We argue that all patients with CAD enrolled in a rehabilitation program should be offered one or several follow-up sessions, and that a maximum exercise testing should preferably be a part of this follow-up. Our data implies that this type of follow-up may be sufficient to prevent deterioration with respect to VO_2peak._ However, follow-up programs of infrequent supervised exercise sessions seemed not to be effective. It is possible that a maintenance program with more frequent supervised exercise could have resulted in improvements in VO_2peak_. In a study of patients with congestive heart failure, Prescott et al. [30] found a small beneficial effect in patients that followed a low-cost maintenance training program with group training sessions every two weeks, indicating that supervised sessions must be more frequent than in our study to result in increased work capacity.

The complete follow-up testing of patients and the high adherence to the supervised exercise are regarded as strengths of our study. Our study is however limited by the fact that the study group consisted of relatively young patients, who probably were quite motivated to exercise without formal follow-up during 12 months. Thus, our results may not be valid for older and less motivated patients with established CAD. Also, neither investigators nor participants were blinded for the study endpoints, which could raise concerns regarding objectivity of testing. However, all patients were told to exercise to complete exhaustion at every exercise test. Further, there was no difference in peak heart rate or peak RER achieved during the baseline and the follow-up test. We also chose to inform the patients in the intervention group about their VO_2peak_ at intermediate tests as we considered it to act motivating for sustained or increase levels of exercise training.

## Conclusions

A 12-month maintenance exercise program consisting of infrequent supervised HIIT sessions, a home-based HIIT program and regular exercise testing did not result in improved adherence to exercise or increased VO_2peak_ in CAD patients compared to usual care. However, both the intervention and the control group sustained their VO_2peak_ 12 months after discharge from formal cardiac rehabilitation.

## Supporting Information

Checklist S1 **CONSORT checklist.**
(DOC)

Protocol S1 **Study protocol.**
(DOC)
