# Supplementary material for: Peak Oxygen Uptake after Cardiac Rehabilitation: A Randomized Controlled Trial of a 12-Month Maintenance Program versus Usual Care
Source: PLoS One. 2014 Sep 23;9(9):e107924. doi: 10.1371/journal.pone.0107924 (PMC4172571; doi:10.1371/journal.pone.0107924)
Supplement: Protocol S1 — Study protocol. (DOC) [file pone.0107924.s002.doc]

## Study protocol:

## Exercise adherence after cardiac rehabilitation

We have previously performed a study comparing the usual care cardiac exercise training program provided by three Norwegian hospitals (St.Olav’s hospital, Levanger Hopsital and Ålesund Hopsital) with high intensity interval training (HIIT) on treadmills after myocardial infarction. We found that patients who did treadmill HIIT had larger improvements in peak oxyen uptake (VO2peak) than subjects randomized to usual care group exercise training . We followed patients for 1,5-3 years after ending the organized exercise training and found that many of the patients quit exercising after the organized program was finished . This has also been shown eaerlier . We know that organized cardiac rehabilitation programs are effective for improving VO2peak in patients with coronary artery disease, but less is known about interventions making the patients continue to exercise after the formal program has ended. A randomized, controlled trial with objective endpoints on the effect of interventions that may increase the adherence to exercise among patients with coronary artery disease is therefore needed . We will therefore perform a study on long-term follow up of patients with coronary artery disease after ended formal cardiac rehabilitation in our health region.

**Short description of the research group**

This study will be a collaboration between Ålesund Hospital, St.Olav’s hospital and the Department of circulation and medical imaging, Norwegian University of Science and Technology. The study adhere to the strategy of the University hospital of further development of medical technological research and the use of basic methods to study clinically well described patient groups. The research group Cardiac Exercise Research Group (H[Uhttp://www.ntnu.no/isb/cerg/U](http://www.ntnu.no/isb/cerg/)H) was established in January 2008 with funds from the Norwegian Research Council, the Norwegian Council on Cardiovascular Disease, St.Olav’s hospital and the Norwegian University of Science and Technology. The group is led by Professor Ulrik Wisløff and the main research focus is to identify cellular and molecular mechanisms behind the benefits of exercise training on the heart, vessels, and skeletal muscles, but also to study the effects of exercise training on survival.

# Aim and hypothesis

What happens to the patients’ VO2peak after ending formal in-hospital rehabilitation? Our hypothesis is that patients who receive written guidelines about exercise training, one monthly supervised exercise training session, a training diary and regular fitness tests (every 3.months), will exercise more and thereby increase their VO2peak more than patients who receive usual care advice given from the hospital staff at discharge from cardiac rehabilitation.

# Methods

This study will assess the effect of regular follow-ups of patients after discharge from two Norwegian Hospitals (St.Olav’s Hospital, and Ålesund Hosptial). The patients will attend usual care cardiac rehabilitation at the three hospitals. At the end of the 12 week training period, they will be offered to participate in the study. Patients that are willing to participate will be randomized to either usual care or a maintenance program after initial testing. Usual care is today advice about being physically active, but with no specific exercise guidelines and without any further follow-up. The maintenance program will consist of a written prescription of how to exercise and an exercise diary. They will be invited to a monthly supervised in-hospital exercise training. They will also be invited to come for a fitness test (with direct measurement of VO2peak) every third month. The maintenance program will last for 12 months. All included patients, both in the maintenance group and in the control group, will be invited back for a follow-up test after one year. The primary endpoint will be change in VO2peak. Secondary outcomes are quality of life, endothelial function and blood markers of cardiometabolic risk.

Peak oxygen uptake will be measured (MetaMax Cortex/Jaeger) during walking on a treadmill. We have a lot of experience in these measurements. The test is continued until subjective exhaustion or clinical symptoms. After completion of the test, the subjects will be asked about their perceived level of exertion according to the Borg scale . At maximum, when ending the test, heart rate will be recorded, as well as the change in heart rate the first minute after stopping (heart rate recovery) while the patient is standing still on the treadmill. We will measure endothelial function by flow-mediated dilatation of the brachial artery. This method is established in our laboratory with an inter observer variability of 0.4% ± 1.3% for the change in artery diameter, and is in accordance with other laboratories. Diameter and flow in the artery is registered at baseline and after a five minutes occlusion of the artery by use of a cuff inflated to 250 mm Hg. Blood flow and artery diameter are measured continuously for four minutes after deflation of the cuff.

## *Participants*

We will include stable coronary artery disease patients 35-80 years old from the middle part of Norway (Trøndelag and Møre & Romsdal counties; St.Olav’s hospital and Ålesund Hospital, respectively). Exclusion criteria: unstable angina pectoris, hemodynamic significant valvular disease (> NYHA class II) or congenital heart disease, chronic obstructive pulmonary disease GOLD stadium III or IV, pregnancy, uncontrolled hypertension, renal failure (creatinine > 140 mmol/L), patients that with a large probability will not complete the study, substance abuse, left ventricle ejection fraction < 30%.

Patients will be stratified according to gender prior to randomization which is done by the Unit for Clinical Research at NTNU. Thus, no person with knowledge about the study is involved in this process. The primary outcome variable is change in VO2peak from baseline (end of formal cardiac rehabilitation) to one year follow-up. Based on data from our previous study in patients with myocardial infarction, we expect participant to decline approximately 2 ml/kg/min with an estimated SD of 4 ml/kg/min with no follow-up (usual care). We anticipate that the maintenance program could result in an increase of 2 ml/kg/min (corresponding to 4 ml/kg/min compared to usual care with SD 4 ml/kg/min), which corresponds to a standardized difference (Δ) = 4/4 =1. We want a statistical power (1-β) = 0.9 and level of significance (α) = 0.05, and therefore needs a minimum of 22 subjects in each group, a total of 44. With a drop-out of 10 %, we aim at including 48 patients in this study.

## *Ethics*

The study is performed according to the Helsinki declaration from 1989. All patients in the study is participating voluntary, and may withdraw consent to participate at any time. All data are treated confidentially, and all personnel involved in the study have confidentiality.

A symptom-limited exercise test may be performed according to Fletcher et al 5-7 days after myocardial infarction. We have a lot of experience with exercise tests in this patient group. All tests will be terminated if the patient reports symptoms of coronary ischemia or if ischemia is found in continuously measured ECG. All departments where the study is performed are covered by the Norwegian Found for Patient Injuries.

# Planned publication

We plan to publish the results from this study in an international journal. We also plan to convey results and knowledge to primary physicians, hospital physicians, and popular science channels such as other journals, TV and/or radio.

**References**

1. Moholdt T, Aamot IL, Granoien I, Gjerde L, Myklebust G, et al. (2012) Aerobic interval training increases peak oxygen uptake more than usual care exercise training in myocardial infarction patients: a randomized controlled study. Clin Rehabil 26: 33-44.

2. Moholdt T, Aamot IL, Granoien I, Gjerde L, Myklebust G, et al. (2011) Long-term follow-up after cardiac rehabilitation A randomized study of usual care exercise training versus aerobic interval training after myocardial infarction. Int J Cardiol 152: 388-390.

3. Gupta R, Sanderson BK, Bittner V (2007) Outcomes at one-year follow-up of women and men with coronary artery disease discharged from cardiac rehabilitation: what benefits are maintained? J Cardiopulm Rehabil Prev 27: 11-18; quiz 19-20.

4. Fletcher GF (2007) Cardiac rehabilitation: Something old--something new--more to do. J Cardiopulm Rehabil Prev 27: 21-23.

5. Borg GA (1982) Psychophysical bases of perceived exertion. Med Sci Sports Exerc 14: 377-381.
